# Metabolomic Profiling Reveals New Insight of Fowl Adenovirus Serotype 4 Infection

**DOI:** 10.3389/fmicb.2021.784745

**Published:** 2022-01-17

**Authors:** Haiying Ma, Yujuan Niu

**Affiliations:** The Biomedical Sciences Institute of Qingdao University (Qingdao Branch of SJTU Bio-X Institutes), Qingdao University, Qingdao, China

**Keywords:** metabolomics, FAdV-4, LMH cells, UHPLC-QTOF-MS, glycolysis, glutamine metabolism

## Abstract

Highly pathogenic fowl adenovirus serotype 4 (FAdV-4) is the causative agent of hydropericardium syndrome (HPS), which is characterized by pericardial effusion and hepatitis, and is one of the foremost causes of economic losses to the poultry industry over the last 30 years. However, the metabolic changes in cells in response to FAdV-4 infection remain unclear. In order to understand the metabolic interactions between the host cell and virus, we utilized ultra-high-performance liquid chromatography/quadrupole time-of-flight tandem mass spectrometry to analyze the metabolic profiles with hepatocellular carcinoma cell line (LMH) infected with FAdV-4. The results showed that FAdV-4 could restore metabolic networks in LMH cells and tricarboxylic acid cycle, glycolysis, and metabolism of purines, pyrimidines, alanine, aspartate, glutamate, and amino sugar and nucleotide sugar moieties. Moreover, FAdV-4 production was significantly reduced in LMH cells cultured in glucose or glutamine-deficient medium. These observations highlighted the importance of host cell metabolism in virus replication. Therefore, similarities and disparities in FAdV-4-regulation of the metabolism of host cells could help improve targeted drug and reduce infection.

## Introduction

Fowl adenovirus serotype 4 (FAdV-4) is a highly pathogenic hepatotropic virus and the etiological agent of hydropericardium syndrome (HPS), which is an infectious disease of broiler chickens characterized by pericardial effusion and acute hemorrhagic hepatitis, resulting in high mortality rates (Schachner et al., 2014; Steer et al., 2015; Zhao et al., 2015; Niu et al., 2018a,b; Wang and Zhao, 2019). Although the diagnostic criteria for HPS are relatively well established, the intrinsic mechanisms underlying FAdV-4 infection of host cells remain unclear.

To elucidate the molecular mechanisms underlying the pathogenesis of FAdV-4 infection, high throughput techniques can be used to reveal global changes in the expression profiles of gene and proteins associated with metabolism and disease progression. Metabolomics is a powerful tool to analyze changes to molecular metabolites caused by viral infection in order to clarify the interactions between host cells and viruses (Lu et al., 2012). Mounting evidence indicates that similarities and disparities in virus-induced regulation of host cell metabolism could help to improve the efficacy of targeted drug therapies and reduce the incidence of infection (Cui et al., 2013; Sun et al., 2013; Ni et al., 2014). However, relatively few studies have investigated changes to host cell metabolomics in response to FAdV-4 infection. The liver, which is one of the main organs involved in metabolism (Han et al., 2016; Sato et al., 2017; Cappel et al., 2019; Zhou et al., 2020), is the target of FAdV-4 infection. Therefore, metabolomics might be useful to elucidate the pathogenic mechanisms underlying FAdV-4 infection of hepatic cells.

However, there are too many different kinds of cells in the liver to simply clarify the metabolomic changes of hepatocytes, so we chose the hepatocellular carcinoma cell line (LMH) which are often employed as an *in vitro* model to study the replication and pathogenesis of FAdV-4 (Niu et al., 2019; Zhao et al., 2020). In our study, ultra-high-performance liquid chromatography/quadrupole time-of-flight tandem mass spectrometry (UHPLC-QTOF-MS) was used to assess regulation of the metabolic network of LMH cells infected with FAdV-4 in order to control the onset and progression of HPS.

## Materials and Methods

### Cell Culture, Virus and Antibodies

Chicken LMH (ATCC^®^ CRL-2117™) were obtained from the American Type Culture Collection (Manassas, VA, United States) and cultured as described in a previous study (Niu et al., 2018a). The FAdV-4 strain (SDDM-4/15) used in the study was prepared as described previously (Niu et al., 2016). Self-prepared rabbit polyclonal antibodies against the FAdV-4 hexon protein were used for indirect immunofluorescence (Niu et al., 2018a).

### Virus Growth Curve

LMH cells at 80% confluence were seeded in the six-well culture plates and infected with FAdV-4 at a multiplicity of infection (MOI) of 1, while mock-infected cells were treated with an equal volume of Dulbecco’s modified Eagle’s medium (DMEM). After 2 h, the inoculum was aspirated and the cells were washed twice with phosphate-buffered saline (PBS). Subsequently, the cells were collected at 12, 24, 36, and 48 h post-infection (hpi). A virus growth curve was constructed based on the data acquired by quantitative real-time polymerase chain reaction (qRT-PCR).

### Sample Preparation

At 24 h before FAdV-4 infection, ∼8.0 × 10^6^ LMH cells were seeded in 100-mm culture plates and cultured to 80–90% confluence. Twelve plates were prepared, six of which were infected with FAdV-4 at an MOI of 1, while the others were used as the mock group. All cells (about 1.2 × 10^7^) were harvested at 24 hpi. Briefly, after the medium was discarded, the cells were washed twice with cold PBS followed by cold 0.9% sodium chloride solution, then quenched with 1 mL of methanol:acetonitrile: water (2:2:1, v/v), and stored at −80°C until metabolomics analysis.

### Metabolite Extraction and Derivatization

After thawing, the cell suspensions were sonicated in an ice bath for 30 min and cooled at −20°C for 10 min before centrifugation at 14,000 × *g* for 20 min at 4°C. Then, the supernatant was evaporated under a stream of nitrogen gas in a vacuum concentrator. Prior to LC-MS/MS analysis, 100 μL of acetonitrile:water solution (1:1, v/v) was added to each tube for resolution and the tubes were swirled and centrifuged at 14,000 × *g* at 4°C for 15 min.

### LC-MS/MS Analysis

The samples were assayed using an Agilent 1290 Infinity UHPLC system (Agilent Technologies, Inc., Santa Clara, CA, United States) and a Triple TOF 6600 mass spectrometer (AB SCIEX, Concord, ON, Canada). Electrospray ionization (ESI) in positive and negative ion modes was used to detect the samples with the following parameters: atomization auxiliary heating gas 1, 60 Psi; auxiliary heating gas 2, 60 Psi; curtain gas, 30 Psi; ion source temperature, 600°C; spray voltage, ± 5,500 V (positive and negative modes); first-grade mass charge ratio detection range, 60–1,000 Da; second grade ion mass charge ratio detection range, 25–1,000 Da; first grade mass spectrum scanning accumulation time, 0.20 s/spectra; and second grade mass spectrum scanning accumulation time, 0.05 s/spectra. The secondary mass spectrum was obtained in data-dependent acquisition mode with a dynamic exclusion of isotopic ion range of 4 Da with scanning and collecting of 10 debris at a time.

### Data Preprocessing and Statistical Analysis

The raw MS data (wiff.scan files) were converted to MzXML files using ProteoWizard MSConvert software before importing into freely available XCMS software. For peak picking, the following parameters were used: centWave m/z, 25 ppm; peakwidth, c (10, 60); and prefilter, c (10, 100). For peak grouping, the following parameters were used: bw, 5; mzwid, 0.025; and minfrac, 0.5. CAMERA (Collection of Algorithms of MEtabolite pRofile Annotation) was used for annotation of isotopes and adducts. In the extracted ion features, only variables with more than 50% of non-zero measurement values in at least one group were retained. Compound identification of metabolites was performed by comparing the accuracies of the m/z values (<25 ppm) and MS/MS spectra with an in-house database established with available authentic standards.

After sum-normalization, the processed data were subjected to multivariate data analysis with the R package “ropls,” including pareto-scaled principal component analysis (PCA), partial least squares discrimination analysis (PLS-DA), and orthogonal partial least-squares discriminant analysis (OPLS-DA). Sevenfold cross-validation and response permutation testing was used to evaluate the robustness of the model. The variable importance in the projection (VIP) value of each variable in the PLS-DA and OPLS-DA models was calculated to indicate potential contribution to the classification. The quality of the models is described by the R^2^X or R^2^Y and Q^2^ values. The Student’s *t*-test was applied to determine the significance of differences between two groups of independent samples. VIP > 1 and a probability (*p*) value < 0.05 were used to screen for significant changes in metabolites. Pearson’s correlation analysis was performed to identify potential correlations between two variables.

### Inhibitory Effects of Related Metabolic Pathways on FAdV-4 Replication

To investigate the impact of host cell metabolism of glucose and glutamine on FAdV-4 replication, LMH cells were cultured in DMEM without glucose or glutamine. As drug treatment samples, FAdV-4-infected LMH cells were treated with 10 or 40 μM 2-deoxy-D-glucose (2dGlc) (a commonly used inhibitor of glycolysis), 1 or 10 μM CB-839, or dimethyl sulfoxide (DMSO) for 48 h. Cells in the DMSO group were infected with FAdV-4 and cultured in DMEM supplemented with glucose and glutamine. Cells were lysed for western blot analysis.

## Results

### Characteristics of FAdV-4 Infected LMH Cells

**FIGURE 1:**
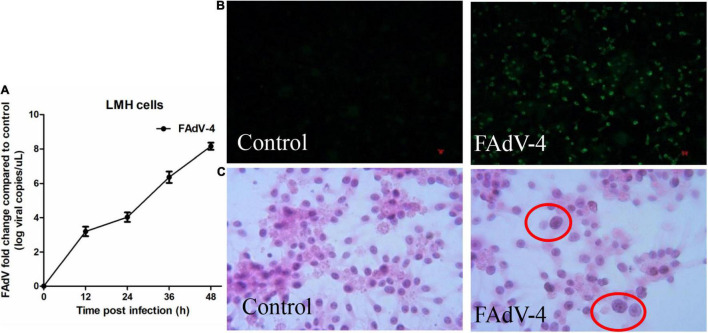
Characteristics of FAdV-4 infected LMH cells. **(A)** The replication characteristics of the virus in LMH cells were determined by qRT-PCR. 50% of the cells were infected with FAdV-4 at 24 hpi by indirect immunofluorescence **(B)**, and viral inclusion bodies were observed in LMH cells (hematoxylin and eosin staining; × 400) **(C)**.

FAdV-4 is known to cause cytopathologic effects in LMH cells. Firstly, the replication characteristics of the virus in LMH cells were determined by qRT-PCR. As shown in Figure 1A, virus titers in LMH cells reached 10^4^ at 24 hpi. Indirect immunofluorescence showed that about 50% of the cells were infected with FAdV-4 at 24 hpi (Figure 1B), and hematoxylin and eosin staining confirmed the formation of viral inclusion bodies (Figure 1C). Combined with previous research results (Niu et al., 2018a), FAdV-4 could cause significant apoptosis, autophagy, and a severe inflammatory response in LMH cells at 24 hpi. Therefore, mock and FAdV-4-infected LMH cells were prepared at 24 hpi for early metabolomics analysis.

### Analysis of Metabolomics Based on LC-MS/MS

**FIGURE 2:**
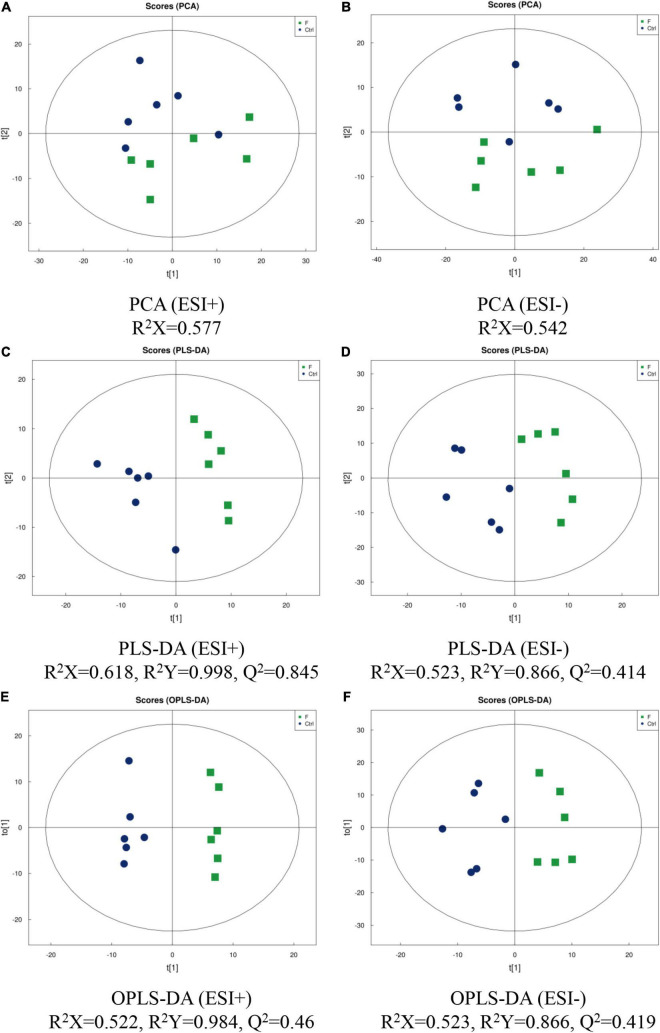
Score plots of PCA, PLS-DA and OPLS-DA of LMH cells infected with FAdV-4. PCA [**(A)** positive ion mode-ESI + ; **(B)** negative ion mode-ESI-], PLS-DA [**(C)** positive ion mode-ESI + ; **(D)** negative ion mode-ESI-], and OPLS-DA [**(E)** positive ion mode-ESI + ; **(F)** negative ion mode-ESI-] models were constructed using LC-MS/MS metabolomics data. Results indicate the separation of control and FAdV-4 group. The ellipses represent 95% confidence intervals of all samples.

The metabolomic profiles of LMH cells infected with FAdV-4 were characterized using LC-MS/MS techniques, and PCA, PLS-DA, and OPS-DA were applied to the data. Analysis of data quality, including total ion chromatogram, PCA, Pearson’s correlation, Hotelling’s t^2^ statistic, multivariate control chart, and relative standard deviation, indicated that the quality of data was acceptable for the following metabolomics analysis. PCA score plots revealed clear separation between the control and FAdV-4-infected groups, under both positive and negative modes (Figures 2A,B). The LC-MS/MS data were further subjected to PLS-DA and OPLS-DA. The quality of the PLS-DA and OPLS-DA models was evaluated based on the parameters R^2^Y and Q^2^, which are measures of fitness and prediction ability, respectively. Generally, Q^2^ is greater than 0.5, indicating that the model is stable and reliable, where 0.3 < Q2 ≤ 0.5 indicates that the model is stable and Q2 < 0.3 indicates low reliability of the model. PLS-DA and OPLA-DA score plots revealed excellent separation of the control and FAdV-4-infected groups (Figures 2C–F).

### Differential Metabolites

**FIGURE 3:**
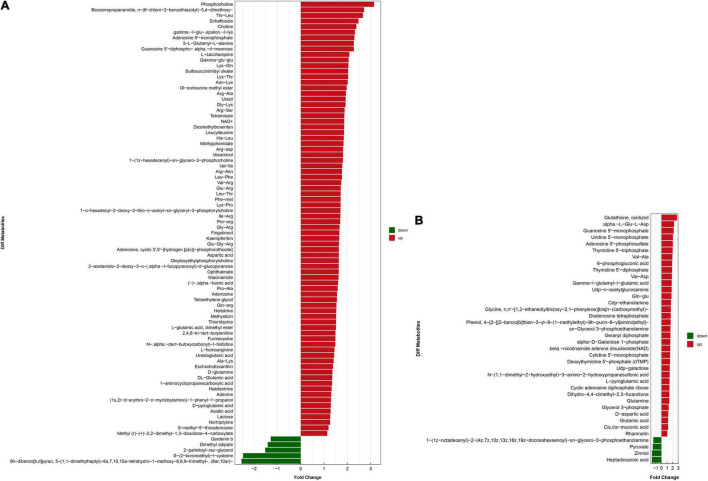
Analysis of the multiple of the difference of metabolite expression in positive ion mode **(A)** and negative ion mode **(B)**. The X-axis indicates the differential expression multiple, red indicates that the differential expression multiple is greater than 1, and green indicates that the differential expression multiple is less than 1. The Y-axis indicates the significant difference metabolites.

OPLS-DA based on a VIP > 1 in and *P* < 0.05 (Student’s *t*-test) revealed significant differences in metabolite screening criteria. The metabolites and associated fold changes in response to FAdV-4 infection of LMH cells are shown in Figure 3 and Tables 1, 2. The differential metabolites, which included 49 amino acids and related derivatives, of LMH cells caused by FAdV-4 infection, included 78 that were increased and five that were decreased in positive ion mode, while 34 were increased and four were decreased in negative ion mode.

**TABLE 1:** Differential metabolites in FAdV-4-infected LMH cells compared to controls in positive ion mode.

Name	VIP	*P*-value	FC (F vs. Ctrl)	KEGG ID
Gly-Arg	2.19	7.79E-07	1.67	
Phe-met	1.02	7.80E-07	1.71	
Pro-arg	1.69	3.49E-05	1.69	
Asn-Lys	1.44	4.11E-05	2.02	
Methylphenidate	1.19	6.48E-05	1.84	C07196
Thr-Leu	1.07	6.63E-05	2.67	
Leucylleucine	1.34	7.61E-05	1.86	C11332
2,4,6-tri-tert-butylaniline	3.71	8.32E-05	1.49	
His-Leu	1.01	9.90E-05	1.85	C05010
Arg-asp	1.13	1.48E-04	1.82	
Pro-Ala	1.03	1.73E-04	1.59	
Leu-Thr	1.17	2.20E-04	1.71	
Leu-Phe	1.94	2.79E-04	1.77	
Arg-Ser	2.01	3.30E-04	1.88	
.gamma.-l-glu-.epsilon.-l-lys	1.45	3.78E-04	2.33	C21730
Lys-Pro	1.36	3.93E-04	1.71	
Glu-Arg	1.68	4.69E-04	1.72	
Ureidoglutaric acid	1.95	4.90E-04	1.43	
Choline	1.33	5.09E-04	2.39	C00114
Adenosine 5′-monophosphate	10.4	5.81E-04	2.31	C00020
Desmethylbosentan	1.54	6.12E-04	1.86	
1-(1z-hexadecenyl)-sn-glycero-3-phosphocholine	1.65	8.45E-04	1.81	
Val-Ile	1.21	9.01E-04	1.79	
Val-Arg	1.46	9.37E-04	1.74	
1-o-hexadecyl-2-deoxy-2-thio-s-acetyl-sn-glyceryl-3-phosphorylcholine	13.5	9.84E-04	1.71	
Nortriptyline	1.03	1.01E-03	1.26	C07274
NAD^+^	2.20	1.02E-03	1.87	C00003
Sulfosuccinimidyl oleate	7.85	1.08E-03	2.03	
Fingolimod	1.34	1.42E-03	1.66	
Eschscholtzxanthin	1.13	1.49E-03	1.38	C08593
Gly-Lys	1.08	1.56E-03	1.92	
Gamma-glu-glu	7.01	1.65E-03	2.06	
Niacinamide	3.88	1.67E-03	1.62	C00153
Benzenepropanamide, n-(6-chloro-2-benzothiazolyl)-3,4-dimethoxy-	2.48	1.74E-03	2.72	
Phosphocholine	8.26	1.75E-03	3.15	C00588
Heliotrine	1.09	1.86E-03	1.53	C10324
Arg-Ala	3.86	1.99E-03	1.94	
Aspartic acid	1.59	2.93E-03	1.64	C00049
Methysticin	6.17	3.10E-03	1.52	C09952
L-saccharopine	1.18	3.40E-03	2.09	C00449
L-glutamic acid, dimethyl ester	1.50	3.44E-03	1.51	
Adenosine, cyclic 3′,5′-[hydrogen [p(s)]-phosphorothioate]	1.31	3.68E-03	1.64	
Ala-Lys	1.85	4.18E-03	1.41	
D-glutamine	4.64	4.22E-03	1.36	C00064
Oleyloxyethylphosphorylcholine	1.62	4.32E-03	1.63	
Schaftoside	5.81	4.46E-03	2.48	C10181
5-L-Glutamyl-L-alanine	1.63	4.60E-03	2.28	C03740
Lactose	4.92	4.71E-03	1.27	C00243
Glu-Gly-Arg	2.90	4.74E-03	1.64	
Lys-Gln	1.73	5.05E-03	2.05	
*S*-(2-boronoethyl)-l-cysteine	1.64	5.59E-03	0.40	
Guanosine 5′-diphospho-.alpha.-d-mannose	1.91	5.69E-03	2.28	C00096
L-homoarginine	1.34	6.45E-03	1.45	C01924
Asiatic acid	1.50	6.67E-03	1.28	C08617
Uracil	1.18	7.46E-03	1.93	C00106
1-aminocyclopropanecarboxylic acid	1.21	7.53E-03	1.35	C01234
Dl-norleucine methyl ester	1.05	8.40E-03	1.97	
(-)-.alpha.-kainic acid	1.76	9.11E-03	1.61	C12819
(1s,2r-d-erythro-2-n-myristoylamino)-1-phenyl-1-propanol	1.14	9.42E-03	1.30	
DL-Glutamic acid	3.29	1.05E-02	1.35	C00025
Tetramisole	1.65	1.08E-02	1.87	
Gardenin b	1.84	1.26E-02	0.78	C15109
Furmecyclox	1.70	1.29E-02	1.49	C18912
2-palmitoyl-rac-glycerol	1.35	1.32E-02	0.66	
2-acetamido-2-deoxy-3-o-(.alpha.-l-fucopyranosyl)-d-glucopyranose	1.39	1.35E-02	1.63	
D-pyroglutamic acid	2.70	1.38E-02	1.29	C02237
Tetraethylene glycol	1.15	1.43E-02	1.56	
6h-dibenzo[b,d]pyran, 3-(1,1-dimethylheptyl)-6a,7,10,10a-tetrahydro-1-methoxy-6,6,9- trimethyl-, (6ar,10ar)-	1.15	1.49E-02	0.39	
Thioridazine	1.61	1.52E-02	1.52	
Ile-Arg	1.83	1.53E-02	1.70	
Vesamicol	1.09	1.83E-02	1.82	
Adenosine	4.76	2.09E-02	1.56	C00212
Lys-Thr	1.05	2.14E-02	2.03	
Adenine	2.54	2.58E-02	1.31	C00147
Halofantrine	1.87	2.71E-02	1.33	C07634
Ophthalmate	2.10	3.06E-02	1.63	C21016
*S*-methyl-5′-thioadenosine	3.63	3.42E-02	1.19	C00170
*N*-.alpha.-(tert-butoxycarbonyl)-l-histidine	3.48	4.22E-02	1.49	
Gln-arg	1.05	4.37E-02	1.53	
Kaempferitrin	1.04	4.45E-02	1.65	C16981
Methyl (r)-(+)-2,2-dimethyl-1,3-dioxolane-4-carboxylate	1.33	4.46E-02	1.14	
Arg-Asn	1.01	4.47E-02	1.77	
Dimethyl adipate	1.74	4.74E-02	0.71	C14570

*Name is the name of the metabolite.*

*FC (fold changes) is the difference multiple.*

*KEGG ID is the KEGG number of the metabolite.*

*E-a represents 10^a in P-value.*

**TABLE 2:** Differential metabolites in FAdV-4-infected LMH cells compared to controls in negative ion mode.

Name	VIP	*P*-value	FC (F vs. Ctrl)	KEGG ID
Val-Ala	1.03	1.01E-04	1.96	
Adenosine 5′-phosphosulfate	5.84	5.41E-04	2.04	C00224
Guanosine 5′-monophosphate	2.03	1.20E-03	2.20	C00144
Deoxythymidine 5′-phosphate (dTMP)	1.14	1.47E-03	1.54	C00364
.beta.-nicotinamide adenine dinucleotide (NAD)	1.04	1.52E-03	1.58	C00003
Gamma-l-glutamyl-l-glutamic acid	3.53	2.69E-03	1.84	C05282
.alpha.-L-Glu-L-Asp	2.92	2.78E-03	2.27	
Uridine 5′-monophosphate	7.41	3.03E-03	2.05	C00105
Cdp-ethanolamine	1.63	4.19E-03	1.71	C00570
Cyclic adenosine diphosphate ribose	2.07	4.53E-03	1.44	C13050
Thymidine 5′-triphosphate	1.78	5.38E-03	2.00	C00459
Val-Asp	1.29	6.41E-03	1.87	
Heptadecanoic acid	1.47	6.91E-03	0.59	
Glutamine	4.79	1.00E-02	1.40	C00064
Diadenosine tetraphosphate	1.02	1.02E-02	1.66	
Geranyl diphosphate	1.67	1.05E-02	1.61	C05847
6-phosphogluconic acid	1.03	1.07E-02	1.91	C00345
Udp-n-acetylglucosamine	12.1	1.14E-02	1.82	C00043
Dihydro-4,4-dimethyl-2,3-furandione	2.30	1.54E-02	1.41	C01125
Gln-glu	2.31	2.18E-02	1.80	
Thymidine 5′-diphosphate	2.13	2.23E-02	1.91	C00363
Pyruvate	1.37	2.30E-02	0.65	C00022
alpha-D-Galactose 1-phosphate	3.09	2.46E-02	1.59	C00103
*N*-(1,1-dimethyl-2-hydroxyethyl)-3-amino-2-hydroxypropanesulfonic acid	1.18	2.66E-02	1.48	
1-(1z-octadecenyl)-2-(4z,7z,10z,13z,16z,19z-docosahexaenoyl)-sn-glycero-3-phosphoethanolamine	2.21	2.85E-02	0.69	
*Cis*, *cis-*muconic acid	9.05	2.90E-02	1.20	C02480
Zinniol	2.73	2.99E-02	0.60	C10840
Glycine, n,n′-[1,2-ethanediylbis(oxy-2,1-phenylene)]bis[n-(carboxymethyl)-	2.60	3.23E-02	1.68	
sn-Glycerol 3-phosphoethanolamine	3.80	3.44E-02	1.62	
D-aspartic acid	3.19	3.51E-02	1.23	C00402
Phenol,4-[2-[[2-benzo[b]thien-3-yl-9-(1-methylethyl)-9h-purin-6-yl]amino]ethyl]-	1.25	3.58E-02	1.65	
Glycerol 3-phosphate	1.35	3.74E-02	1.30	C00093
Udp-galactose	2.14	3.81E-02	1.50	C00052
Cytidine 5′-monophosphate	1.25	3.92E-02	1.55	C00055
Rhamnetin	2.63	4.18E-02	1.05	C10176
L-pyroglutamic acid	3.53	4.48E-02	1.45	C01879
Glutathione, oxidized	9.09	4.76E-02	2.78	C00127
Glutamic acid	4.00	4.99E-02	1.20	C00025

*Name is the name of the metabolite.*

*FC (fold changes) is the difference multiple.*

*KEGG ID is the KEGG number of the metabolite.*

*E-a represents 10^a in P-value.*

### Changes to Metabolic Pathways

**FIGURE 4:**
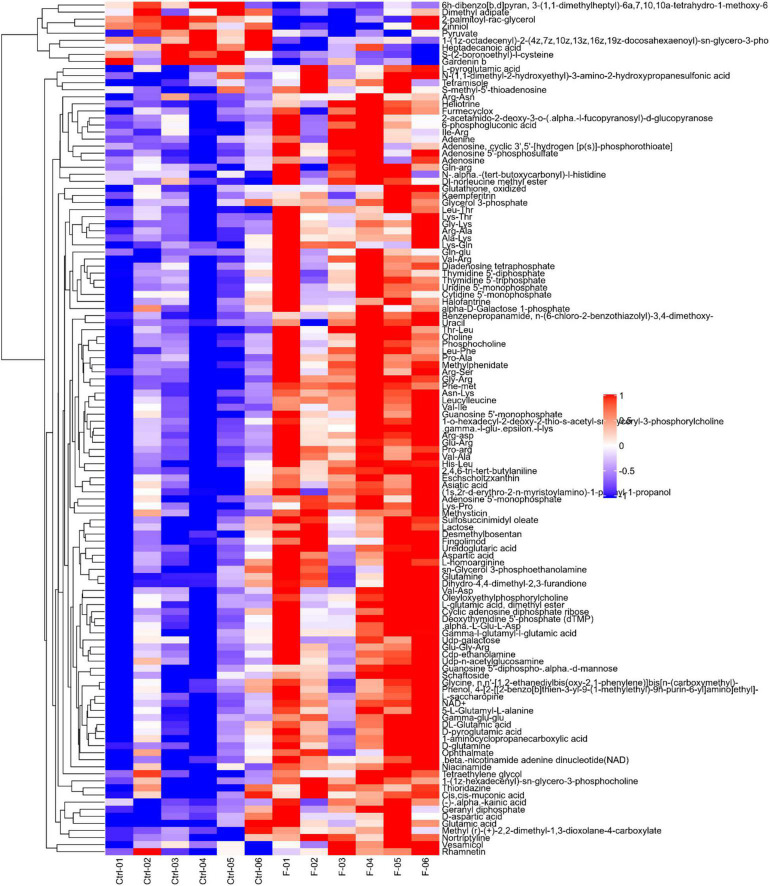
Heatmap of differentially expressed metabolites in LMH cells infected with FAdV-4. Each row in the figure represents a differential metabolite (the ordinate is the metabolite with significant differential expression), and each column represents a group of samples (the abscissa is the sample information). Red represents significant up regulation, blue represents significant down regulation, color depth indicates the degree of up regulation and down regulation, and metabolites with similar expression patterns gather in the same cluster on the left.

**FIGURE 5:**
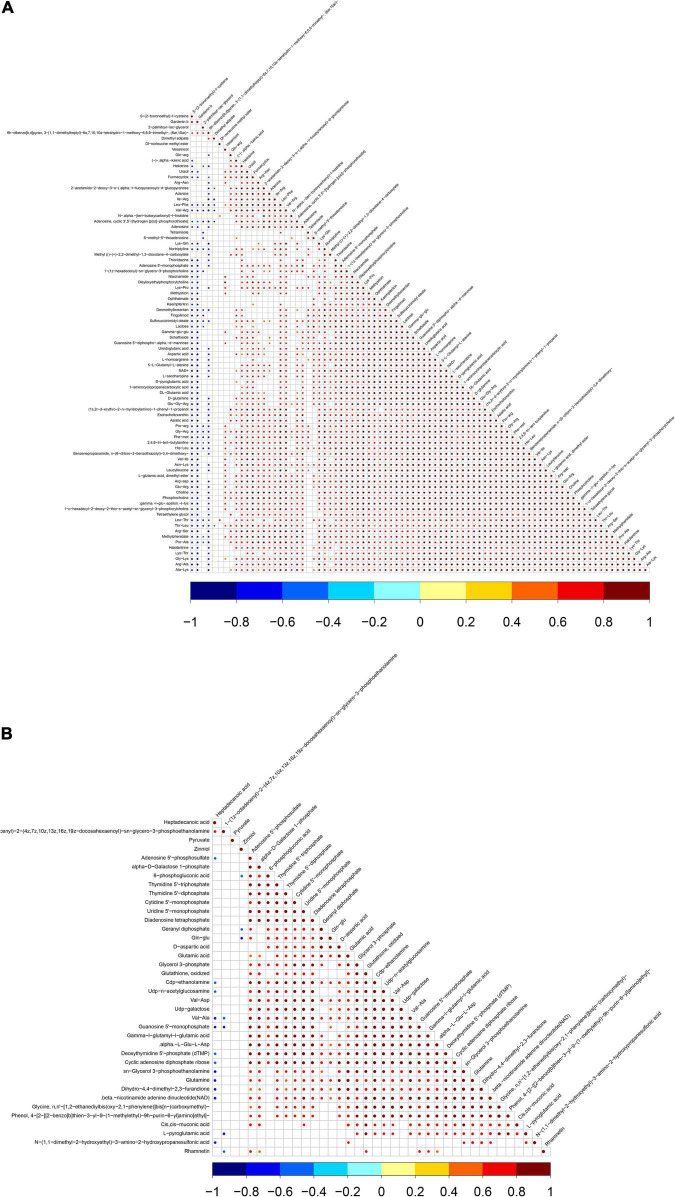
Pearson’s of differentially expressed metabolites in positive ion mode **(A)** and negative ion mode **(B)**. Red indicates positive correlation, blue indicates negative correlation, and white indicates no significant correlation. The color depth is related to the absolute value of correlation coefficient. The higher the degree of positive correlation or negative correlation, the darker the color. The size of the point is related to the significance of the correlation, the more significant.

**FIGURE 6:**
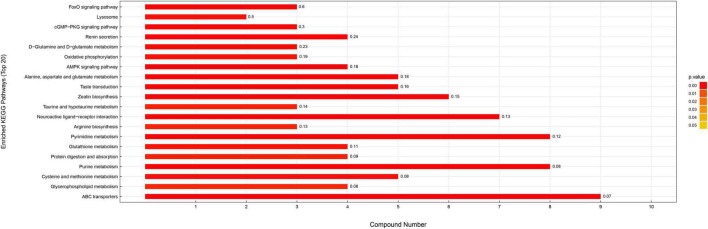
KEGG enrichment pathway map. The vertical axis represents each KEGG metabolic pathway, and the horizontal axis represents the number of differentially expressed metabolites in each KEGG metabolic pathway. Color represents the *P* value of enrichment analysis. The darker the color is, the smaller the *P* value is, and the more significant the enrichment degree is. The number on the column represents the proportion of different metabolites in the detected metabolites.

**FIGURE 7:**
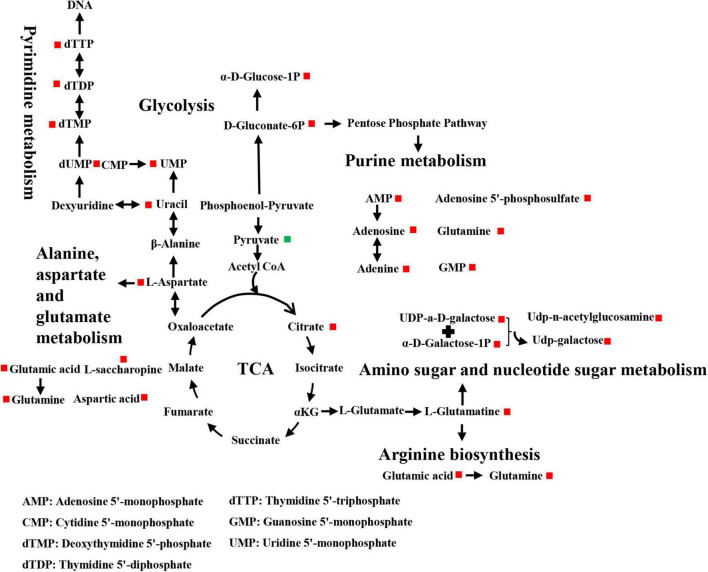
Schematic representation of altered metabolic pathways in LMH cells infected with FAdV-4.

Differential metabolites were further analyzed to explore the effects of FAdV-4 infection on the metabolic pathways of LMH cells. The relationships among the differential metabolites were investigated using heatmap analysis (Figure 4) and Pearson’s correlation (Figure 5). The results indicated that metabolites in the same or related metabolic pathways were closely connected. When the significance level of metabolite enrichment in each pathway was analyzed and calculated with the Fisher’s exact test, 60 pathways were potentially changed in LMH cells infected with FAdV-4 (Table 3). The top 20 metabolic pathways with the highest significance were selected based on *P* values and presented in the form of a histogram (Figure 6). The top 20 metabolic pathways mainly involved pyrimidine metabolism, purine metabolism, amino acid metabolism, carbohydrate metabolism, glutathione metabolism, and cofactor and vitamin metabolism, in addition to some signal transduction pathways. Next, some metabolites with significant differences were further screened to establish connections among the related metabolic pathways (Figure 7). The results showed that the tricarboxylic acid (TCA) cycle, purine metabolism, pyrimidine metabolism, alanine, aspartate, and glutamate metabolism, amino sugar and nucleotide sugar metabolism, and glycolysis were significantly up-regulated and closely connected.

**TABLE 3:** Differential metabolites in FAdV-4-infected LMH cells compared to controls in negative ion mode.

Metabolic pathway	*P*-value
Pyrimidine metabolism	0.00
Neuroactive ligand-receptor interaction	0.00
Zeatin biosynthesis	0.00
Purine metabolism	0.00
Alanine, aspartate and glutamate metabolism	0.00
FoxO signaling pathway	0.00
ABC transporters	0.00
Taste transduction	0.00
Renin secretion	0.00
AMPK signaling pathway	0.00
cGMP-PKG signaling pathway	0.00
D-Glutamine and D-glutamate metabolism	0.00
Cysteine and methionine metabolism	0.00
Oxidative phosphorylation	0.00
Lysosome	0.00
Glutathione metabolism	0.00
Taurine and hypotaurine metabolism	0.00
Protein digestion and absorption	0.00
Arginine biosynthesis	0.00
Glycerophospholipid metabolism	0.00
Aminoacyl-tRNA biosynthesis	0.00
Metabolic pathways	0.00
Biosynthesis of amino acids	0.00
Nicotinate and nicotinamide metabolism	0.00
Olfactory transduction	0.00
Longevity regulating pathway	0.00
Glutamatergic synapse	0.00
GABAergic synapse	0.00
Pantothenate and CoA biosynthesis	0.00
Longevity regulating pathway—worm	0.01
beta-Alanine metabolism	0.01
Calcium signaling pathway	0.01
Photosynthesis	0.01
Insulin secretion	0.01
Synaptic vesicle cycle	0.01
Monobactam biosynthesis	0.01
Regulation of lipolysis in adipocytes	0.01
Platelet activation	0.01
Bacterial secretion system	0.01
HIF-1 signaling pathway	0.01
Galactose metabolism	0.02
Proximal tubule bicarbonate reclamation	0.02
Histidine metabolism	0.02
Glycine, serine and threonine metabolism	0.02
Nitrogen metabolism	0.02
Biosynthesis of various secondary metabolites—part 3	0.02
Two-component system	0.02
Aldosterone synthesis and secretion	0.03
Carbon fixation in photosynthetic organisms	0.03
Thermogenesis	0.03
cAMP signaling pathway	0.03
Glyoxylate and dicarboxylate metabolism	0.03
Amino sugar and nucleotide sugar metabolism	0.03
Glucagon signaling pathway	0.03
Carbon metabolism	0.04
Ferroptosis	0.04
mTOR signaling pathway	0.04
PI3K-Akt signaling pathway	0.04
Thiamine metabolism	0.05
Glycolysis/Gluconeogenesis	0.05

*0.00 means that P-value is less than 0.01.*

### Glucose and Glutamine Metabolism on FAdV-4 Replication

**FIGURE 8:**
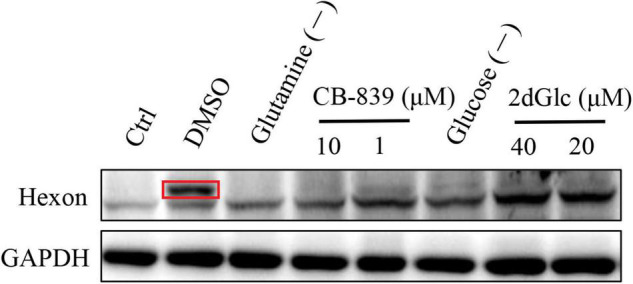
Western blot analysis hexon expression in LMH cells by affecting glucose and glutamine metabolism. The destination strip is marked in the red box, and the below bands are non-specific.

As shown in Figure 8, treatment of LMH cells with either glutamine withdrawal or 1 or 10 μM CB-839 significantly reduced FAdV-4 replication and lowered viral yields relative to DMSO treatment in the presence of 4 mM glutamine. Moreover, the inhibitory effect of CB-839 on FAdV-4 was dose-dependent. FAdV-4 production was significantly reduced in LMH cells cultured in glucose-deficient medium. Treatment of FAdV-4-infected cells with 20 or 40 μM 2dGlc also effectively impaired the production of infectious particles.

## Discussion

Viruses, as obligate intracellular parasites, are completely dependent on host cell metabolism for replication, production, and release (Harries et al., 2010; Thaker et al., 2019b). In order to fulfill these requirements, viruses have evolved different mechanisms to reprogram and exploit the host metabolism (Mesquita and Estaquier, 2018). Therefore, understanding changes to the metabolism of virus-infected host cells would be useful to develop targeted therapies. In recent years, metabolomics analysis has been conducted of various viruses, such as Zika virus (Thaker et al., 2019a), Newcastle disease virus (Liu et al., 2019), and classical swine fever virus (Gou et al., 2017). This study is the first to employ metabolomics to analyze changes in metabolites and metabolic pathways in FAdV-4-infected LMH cells, which revealed insights into the mechanisms of the pathogenesis and host cell interactions of FAdV-4.

The results of this study provide useful information on alterations to metabolites and associated pathways in LMH cells infected with FAdV-4 (Figures 3, 4, 6). Several pathways involved in energy metabolism were found to be perturbed during FAdV-4 infection, including the TCA, purine metabolism, pyrimidine metabolism, glycolysis, and the metabolism of some amino acids. In a sense, changes to these metabolic pathways could reflect the contributions of host cells to virus proliferation.

Glucose oxidation is a major source of carbon and energy in cellular bioprocesses. During glycolysis, glucose is metabolized to pyruvate (Tohyama et al., 2016). In the presence of oxygen or under oxygen-limiting conditions, pyruvate is catabolized in the TCA to generate large amounts of adenosine triphosphate (ATP) or lactate. Increased amounts of glycolytic intermediates provide the precursors required for synthesis of nucleotides, amino acids, and lipids, as well as cellular redox homeostasis (Vander Heiden et al., 2009; DeBerardinis and Chandel, 2016). Metabolomics analysis revealed that FAdV-4 significantly affected glycolysis and, subsequently, the intermediates of purine metabolism. However, FAdV-4 infection did not induce significant changes to the intermediates of the TCA cycle for ATP production, with the exception of citrate. In a previous study, FAdV-4 infection induced swelling of the mitochondria of hepatocytes and the disappearance of cristae. Oxidative phosphorylation and ATP synthesis occur in the mitochondria (Zorova et al., 2018). However, FAdV-4 infection impairs oxidative phosphorylation, at least to a certain extent, which can result in mitochondrial injury. On the other hand, pyruvate carboxylation will replenish the metabolites involved in the TCA cycle, while redirecting other TCA intermediates to pyrimidine biosynthesis (Vastag et al., 2011). In this study, pyrimidine biosynthesis was increased (Figures 6, 7). The metabolism of purines and pyrimidines can be summarized as nucleic acid metabolism. Increase metabolism of purines and pyrimidines provides materials for virus replication. In general, glycolysis is a key metabolic pathway. Previous studies have shown that glucose metabolism can affect virus replication (Yu et al., 2011; Thai et al., 2014; Kong et al., 2015). The results showed that in glucose-deficient medium, FAdV-4 production was reduced in LMH cells. Later studies showed that treatment of FAdV-4-infected cells with 2dGlc, a commonly used inhibitor of glycolysis, also effectively impaired the production of infectious particles (Figure 8), likely due to reduced glycosylation of viral glycoproteins. Although it is clear that glucose metabolism is important for FAdV-4 infection, the exact mechanisms responsible for virus-induced activation of glycolysis are still not fully understood. Since glucose uptake is important for bioenergy requirements and cell biomass, increased glycolysis may be used by viruses as a source of biomass replication. Increasing glucose uptake may also be required for other metabolic pathways, such as the pentose phosphate and nucleic acid pathways. In fact, virus-induced changes to glucose metabolism seem to play a key role in successful infection, thus it is important to understand the precise molecular mechanisms that drive this reprogramming.

Apart from glucose, glutamine, which is classified as a non-essential amino acid, plays a crucial role in cell proliferation. Virus infection of cells will enhance bioenergetics and macromolecular synthesis, which may include glutamine uptake and utilization. Glutamine serves as a carbon source to support the biosynthesis of lipids, amino acids, and nucleotides (Lunt and Vander Heiden, 2011; DeBerardinis and Chandel, 2016). Therefore, glutamine availability is essential for the replication of several viruses. In this study, glutamine metabolism was notably increased in response to FAdV-4 infection (Figures 6, 7). Moreover, FAdV-4 replication was found to be glutamine-dependent as cultivation in glutamine-free medium completely abolished the production of infectious viral particles. Treatment of LMH cells with 1.0 or 10 μM CB-839 significantly decreased replication of FAdV-4 as compared with DMSO treatment (Figure 8). This observation highlights the importance of glutamine in virus replication, although future studies are needed to further clarify the underlying molecular mechanisms.

FAdV-4 infection caused changes to a variety of metabolic pathways, among which the metabolism of purines and pyrimidines is particularly important. It is a known that virus infection affects nucleotide metabolism of the host cells in order to meet the nucleic acid needs of the virus (Munger et al., 2006; Vastag et al., 2011; Thai et al., 2014). In this study, the concentrations of the intermediates of the purine and pyrimidine biosynthesis pathways had increased (Figure 7).

In summary, these metabolomics data provide evidence that FAdV-4 restored the metabolic networks in LMH cells. Various metabolic pathways, including glycolysis and the metabolism of glutamine, amino acids, purines, and pyrimidines, were altered. Hence, virus infection modified the metabolism of the host cell to provide energy and materials for replication. The identification of these changes will provide considerable important information for further understanding of FAdV-4 replication, pathogenesis, and drug sensitivity.

## Data Availability Statement

The original contributions presented in the study are included in the article/supplementary material, further inquiries can be directed to the corresponding author/s.

## Author Contributions

YN contributed to design of the study and revised the manuscript. HM drafted the manuscript. Both authors contributed to the article and approved the submitted version.

## Conflict of Interest

The authors declare that the research was conducted in the absence of any commercial or financial relationships that could be construed as a potential conflict of interest.

## Publisher’s Note

All claims expressed in this article are solely those of the authors and do not necessarily represent those of their affiliated organizations, or those of the publisher, the editors and the reviewers. Any product that may be evaluated in this article, or claim that may be made by its manufacturer, is not guaranteed or endorsed by the publisher.
